# The prevalence of depression among patients with tuberculosis: a systematic review and meta-analysis

**DOI:** 10.1186/s12991-020-00281-8

**Published:** 2020-05-07

**Authors:** Bereket Duko, Asres Bedaso, Getinet Ayano

**Affiliations:** 1 Faculty of Health Sciences, College of Medicine and Health Sciences, Hawassa University, Hawassa, Ethiopia; 2 School of Public Health, Curtin University, Perth, Australia; 3 Research and Training Department, Amanuel Mental Specialized Hospital, Addis Ababa, Ethiopia

**Keywords:** Prevalence, Depression, TB, Systematic review, Meta-analysis

## Abstract

**Background:**

Evidence has shown that the prevalence of depression is much higher among patients with tuberculosis (TB) and this, in turn, may adversely impact compliance with anti-TB medications. Therefore, this systematic review and meta-analysis aimed to quantitatively summarize epidemiologic evidence on the prevalence of depression among patients with TB and formulate a recommendation for future clinical practice as well as research.

**Methods:**

We followed the preferred reporting items for systematic reviews and meta-analyses (PRISMA) guidelines to conduct this review. We searched PubMed, EMBASE, SCOPUS and Psych INFO to identify relevant studies that investigated the prevalence of depression among TB patients. We also supplemented our electronic search with manual searching to include all pertinent studies in the analysis. We used a Comprehensive Meta-Analysis software version 3.0 (CMA 3.0) to conduct a meta-analysis. We conducted a subgroup and sensitivity analysis and Cochran’s *Q*- and the *I*^2^-statistics were used to assess heterogeneity. The evidence for the presence of publication bias was checked by using Egger’s test and visual inspection of the symmetry in funnel plots.

**Results:**

We identified a total of 25 studies that included 4903 participants across seven countries. In our analysis, the pooled estimated prevalence of depression among TB patients was found to be 45.19% (95% CI 38.04–52.55). The prevalence was higher in MDR-TB 52.34% (95% CI 38.09–66.22) than non-MDR-TB 43.47% (95% CI 35.88–51.37) patients. We also found that the pooled prevalence of depression was higher among females 51.54% (95% CI 40.34–62.60) when compared to males 45.25% (95% CI 35.19–55.71). The pooled prevalence of depression was 45.45% as measured by HRDS, and it was 55.62%, 45.52%, and 38.36% as measured by BDI, HADS and PHQ-9, respectively.

**Conclusion:**

Our finding suggested that the pooled estimated prevalence of depression among tuberculosis patients was relatively high. Screening and management of depression among TB patients were warranted to alleviate suffering. Moreover, the integration of tuberculosis program with regular psychiatry services may substantially reduce the burden.

## Background

Tuberculosis (TB) a chronic infectious disease, which is caused by *Mycobacterium tuberculosis* (MTB) bacteria, affects the lungs and other parts of the body [1]. The World Health Organization (WHO) report of 2019 showed that approximately 10 million people fell ill with TB, 1.5 million people died of TB and 484,000 people fell ill with drug-resistant TB in 2018 [2]. On the other hand, 58 million people lives saved between 2000 and 2018 by efforts to end tuberculosis globally [2]. However, the high burden of TB on morbidity and mortality constitutes a significant concern in low and middle-income countries [2, 3]. Numerous risk factors have been associated with mortality that resulted from poor anti-TB medication adherence. Poor compliance with medications may lead to treatment default and result in severe medical complications, for example, multidrug-resistant TB (MDR-TB) [4].

Depression is among common mental health problems that occur in patients with tuberculosis, characterized by persistent depressed mood, lack of pleasure in everyday activities, reduced energy, vegetative symptoms, suicidal ideation and attempt and causing varying levels of social and occupational dysfunctions [5]. Depression is a major contributor to the overall global burden of disease and affected more than 264 million people of all ages in 2019 [6]. Findings from different studies showed that approximately 800,000 people die due to suicide every year and over 50% of all people who die by suicide suffer from major depression [7, 8]. Evidence from the epidemiological study also showed that the lifetime risk of suffering from depression among the general population is 7.2% and 4.4% in females and males, respectively [9].

Depression and tuberculosis are often coexisting in individuals. They share common risk factors, which suggest the high magnitude of their comorbidity as reported by different studies to range from 10 to 52% [10, 11]. For example, the increase of pro-inflammatory cytokines characteristic of depression leads to reduced activation of the cellular and humoral immune systems and this, in turn, contributes to the progression of tuberculosis [11]. Similarly, infection resulted from tuberculosis can cause chronic inflammation, releasing pro-inflammatory cytokines that stimulate enzymes functioning at the central nervous system and also some of the anti-TB medications may play a role in mental health problems such as depression [12]. When depression is comorbid with tuberculosis, it will lead to poor quality of life, lack of adherence to anti-Tb medications, progressions to MDR-TB and finally end up with mortality resulting from the disease [13, 14].

Epidemiological findings from different studies showed different rates of prevalence of depression among patients with tuberculosis [15–19]. For example, a cross-sectional study that assessed the prevalence of depression among tuberculosis patients in Pakistan reported 47.2% [15]. In contrast, another cross-sectional study from Greece that assessed the prevalence of depression in patients with bronchial asthma, chronic obstructive pulmonary disease, and tuberculosis in a general hospital of chest diseases reported 9.93% [16]. The disparities in the rates of prevalence may be attributed to variations in the assessment tools of depression among TB patients.

A systematic review and meta-analysis was conducted in 2018 to quantify mental health problems such as depression, anxiety, and psychosis as well as the health-related quality of life in patients with multidrug-resistant tuberculosis (MDR-TB) [20]. This review included a total of 32 studies that assessed mental health problems including substance use disorders among MDR-TB patients across 20 countries and estimated the prevalence of mental health problems at baseline and after MDR-TB treatment initiation. This study reported that the pooled estimated prevalence of depression, anxiety, and psychosis among MDR-TB patients was 50%, 16%, and 4%, respectively, before initiation of the tuberculosis medications. However, our review focuses on the prevalence of depression among tuberculosis patients irrespective of the severity of tuberculosis (MDR-TB or non-MDR-TB). Therefore, this study aimed to quantitatively summarize the prevalence of depression among TB patients and formulate a recommendation for future clinical practice as well as research.

## Methods

### Search strategy

We performed an extensive search of literature as suggested in the guideline of reporting systematic review and meta-analysis (PRISMA) [21]. We have reviewed both published and gray literature on the prevalence of depression among patients with tuberculosis using the following major databases: PubMed, SCOPUS, EMBASE, and Psych INFO. All published and unpublished articles up to December 2019 were included in the systematic review and meta-analysis. We conducted our search in PubMed using the following terms: “depression OR depressive disorder OR major depression OR depressive symptoms OR mental health problems OR distress OR psychological distress AND tuberculosis OR TB OR MDR-TB OR respiratory disease AND prevalence OR magnitude OR epidemiology OR incidence”. We used specific-subjects headings for EMBASE and SCOPUS database searching. Besides, we have manually searched the reference lists of eligible articles.

### Eligibility criteria

Evaluation of the relevant studies using their title and abstract was done before the retrieval of full-text articles for further screening, the two reviewers (BD and GA). A predefined inclusion and exclusion criteria were employed to screen the retrieved full articles and any disagreement during the process was solved via discussion with a third reviewer (AB). Cross-sectional and other observational studies that assessed the prevalence of depression among patients with tuberculosis (MDR-TB and non-MDR-TB patients) and published in the English language were included in the review. Duplicate studies, commentaries, reviews, letters, editorials, and short communications were excluded from the review.

### Methods for data extraction and quality assessment

A predesigned standardized data extraction form was utilized to extract data from the studies included in the systematic review and meta-analysis. We have extracted the following information from each study: the name of the first author, publication year, study setting, study design, sample size, the prevalence of depression and data measurement tools used for assessing depression. Two reviewers extracted the data from the included studies independently and any disagreements raised during data extraction were resolved through discussion with a third reviewer. A modified version of the Newcastle–Ottawa Scale (NOS) [22] was used to evaluate the quality of the included studies. An ascertainment of depressive symptoms, statistical quality, sample representativeness, sample size and comparability between participants were the domains NOS scale to assess the quality of individual studies.

### Data synthesis and analysis

We used a comprehensive meta-analysis software version 3.0 (CMA 3.0) to conduct this meta-analysis. The random effect meta-analysis model was used to estimate the overall pooled estimated prevalence of depression among patients with tuberculosis. The *Q*- and *I*^2^-statistics were used to check the heterogeneity among the studies included in the review [23]. The magnitude of statistical heterogeneity between studies was assessed using *I*^2^-statistic and values of 25, 50 and 75% were considered to represent low, medium and high, respectively [24, 25]. We have used subgroup and sensitivity analysis to explore the potential source of heterogeneity. The data assessment instrument that was used to assess depression, types of tuberculosis (MDR-TB and non-MDR-TB), the gender of the study participants and the quality of studies were used to determine the possible source of heterogeneity between the studies. The funnel plot and Egger’s regression tests were checked to assess publication bias.

## Results

### Identification of studies

**Fig. 1:**
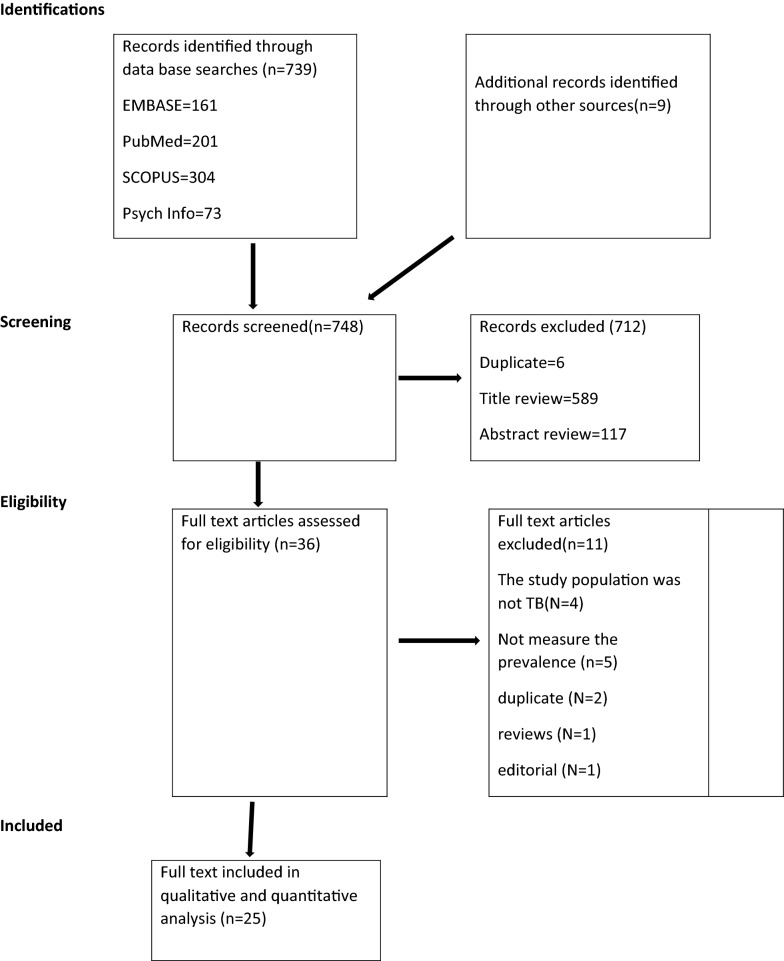
PRISMA flowchart of review search

Our electronic search engine and strategies resulted in a total of 739 articles. Additionally, we identified 9 articles by our manual search making the total articles of 748. Of these, 595 were excluded during the evaluation of duplicate and titles as they did not meet the inclusion criteria. Our appraisal of abstract resulted in the exclusion of a further 117 articles. Therefore, a full-text of 36 studies were retrieved for further evaluation and 11 of these were excluded (Fig. 1).

### Characteristics of included studies

In this review, a total of 25 studies were included in the final meta-analysis conducted in seven countries representing 4903 participants. The characteristics of the study populations included in this study are depicted in Table 1. Out of 25 studies included in the review, seven were from Pakistan [15, 26–31], seven from India [32–38], three from Nigeria [18, 39, 40], four from Ethiopia [11, 19, 41, 42], one from Brazil [43], one from China [44], one from Cameron [45], and one from Turkey [47]. The studies included in this review were published between 2006 and 2019, with the sample size ranging between 45 participants in India and 1252 participants in China. Depression among TB patients was predominantly measured using the PHQ-9 scale. The PHQ-9 was used in 12 studies, HADS in 7 studies, BDI in 3 studies, and the HDRS in 3 studies.

**Table 1:** Characteristics of studies included in the systematic review and meta-analysis

Study name	Country	Sample size	Type of TB	Year data collected	Depression assessment instrument	Prevalence in male (%)	Prevalence in female (%)	Overall prevalence	NOS quality score
Aamir et al. [26]	Pakistan	65	MDR-TB	2007–2008	HADS			36.9	Low
Anwar et al. [27]	Pakistan	60	TB	2009	BDI-II	86.1	70.8	80	Low
Javald et al. [28]	Pakistan	289	MDR-TB	2012–2013	HDRS	81.3	92.9	69.55	High
Ahmed et al. [29]	Pakistan	83	TB	2015	HDRS			49.4	Low
Hussain et al. [15]	Pakistan	108	TB	2007	HADS	50	45.3	46.3	Moderate
Amreen et al. [29]	Pakistan	100	TB	2014	PHQ9			56	Moderate
Mehreen et al. [30]	Pakistan	213	MDR-TB	2013	HDRS			65.5	High
Yilmaz et al. [46]	Turkey	208	TB	2014–2015	HADS	60.3	61.0	60.5	Moderate
Ravi et al. [32]	India	120	TB	2017–2018	PHQ9	50	47.4	49	Moderate
Kumar et al. [33]	India	100	TB	2015	BDI-II			35	Low
Basu et al. [34]	India	110	TB	2012	PHQ9	72	72.7	61.8	Moderate
Dahiya et al. [35]	India	106	TB	2016	BDI-II	42.86	77.3	50	Moderate
Arjun et al. [36]	India	200	TB	2009–2011	HDRS	39.8	39.1	39.5	Moderate
Chandra et al. [37]	India	100	MDR-TB	2014–2015	HADS	56.3	52.8	55	Moderate
Mrinalini et al. [38]	India	100	TB/HIV	2012–2014	PHQ9			16	Moderate
Dos et al., 2016 [43]	Brazil	86	MDR-TB	2013	HADS	28.3	46.2	31.4	Low
Wang et al. [44]	China	1252	TB	2014–2015	PHQ9	18.4	16.76	17.7	High
Baba et al. [39]	Nigeria	65	TB	2008	PHQ9	29.3	25	27.7	Low
Larson et al. [40]	Nigeria	371	TB/HIV	2013–2015	PHQ9	29.9	26.9	30	High
Ige et al. [18]	Nigeria	88	TB	2010	HDRS	20	57.1	45.5	Low
Kehbila et al. [45]	Cameroon	265	TB	2015	PHQ9	22.6	38.2	61.1	High
Duko et al. [19]	Ethiopia	417	TB	2014	HADS	43.8	43.2	43.4	High
Ambaw et al. [11]	Ethiopia	657	TB	2015	PHQ9	49.7	58.1	54	High
Dasa et al. [41]	Ethiopia	403	TB	2017	PHQ9			51.9	High
Molla et al. [42]	Ethiopia	415	TB	2018	PHQ9	39.6	51.9	31.1	High

### Quality of included studies

The Newcastle–Ottawa scale (NOS) scale with slight modifications was used to evaluate the quality of studies included in the review. Of the included studies, nine studies were high quality (NOS score 8 and above), nine moderate quality (NOS score between 6 and 7 inclusive) and seven were low-quality studies (NOS score less than or equal to 5) (Table 1).

### Meta-analysis

#### The prevalence of depression among patients with tuberculosis

**Fig. 2:**
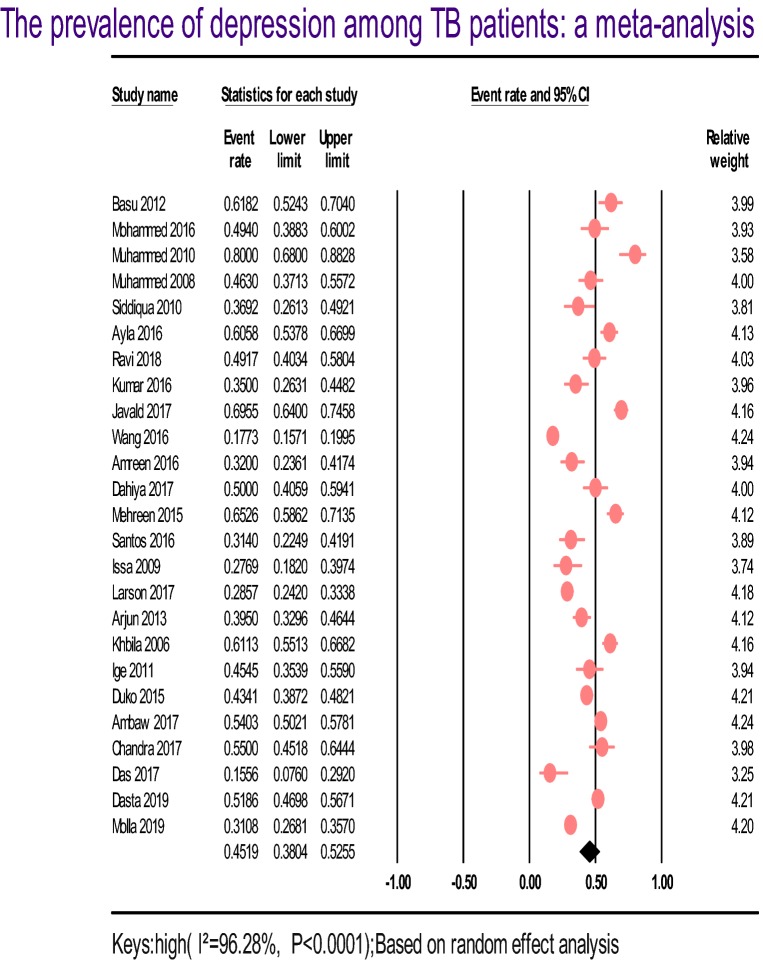
Forest plot of the prevalence of depression among TB patients: a meta-analysis

Twenty-five studies that reported the prevalence of depression among patients with tuberculosis were included in the final analysis (Table 1). Based on the results of the random-effects meta-analysis model, the pooled estimated prevalence of depression among patients with tuberculosis was 45.19% (95% CI 38.04–52.55). We found significant heterogeneity for this analysis (*I*^2^ = 96.28%; *p* < 0.001) (see Fig. 2).

### Subgroup analysis

#### The prevalence of depression in patients with MDR-TB and non-MDR-TB

In this review, five of the studies reported the prevalence of depression in MDR-TB whereas 20 of them reported the prevalence of depression in non-MDR-TB cases. In the analysis of those studies which reported prevalence of depression among MDR-TB and non-MDR-TB cases, we found that the prevalence of depression was higher in MDR-TB 52.34% than non-MDR-TB 43.47%. A significant heterogeneity was found in both MDR-TB (*I*^2^ = 92. 55; *p* < 0.001) and non-MDR-TB (*I*^2^ = 99.26%; *p* < 0.001) (see Table 2).

**Table 2:** Sensitivity analysis of all studies based on type of TB, instrument used, and study quality of the included studies

Subgroups	Studies, *n*	Prevalence (%)	95% CI	Heterogeneity between groups (*p* value)
Type of TB				
MDR-TB	5	52.34	38.09–66.22	0.320
Non-MDR TB	20	43.47	35.20–52.65	
Instrument used				
BDI	3	55.62	32.00–76.94	0.073
HADS	7	46.52	39.25–53.94	
HDRS	3	61.09	48.47–72.38	
PHQ-9	12	38.36	28.52–49.26	
Quality of studies				
High	9	46.06	33.15–58.52	0.657
Moderate	9	47.85	40.62–55.16	
Low	7	40.54	27.82–54.68	

*MDR*-*TB* multidrug-resistant tuberculosis, *BDI* Beck Depression Inventory, *HADS* The, Hospital Anxiety and Depression Scale, *HDRS* Hamilton Depression Rating Scale, *PHQ*-*9* Patient Health Questionnaire-9

### Subgroup analysis of the prevalence of depression among TB patients by the assessment instrument

We also conducted a subgroup analysis by the type of assessment instruments used to measure depression. The pooled prevalence of depression was 45.45%, 55.62%, 45.52% and 38.36% for studies that assessed depression using HRDS, BDI, HADS and PHQ-9, respectively. The heterogeneity was significant for all studies performed by BDI (*I*^2^ = 79.38%, *p* < 0.0001), HADS (*I*^2^ = 80.40%, *p* < 0.0001), HRDS (*I*^2^ = 87.87%, *p* < 0.0001) as well as PHQ-9 (*I*^2^ = 97.63%, *p* < 0.001) (see Table 2).

### Subgroup analysis of the prevalence of depression among TB patients by gender of participants

In this review, 17 of the studies reported the prevalence of depression in male and female participants. Analysis of those studies which reported prevalence of depression in males and females, we found that the prevalence of depression was higher in females 51.54% (95% CI 40.34–62.60) than males 45.25% (95% CI 35.19–55.71). A significant heterogeneity was found in both females (*I*^2^ = 92. 55; *p* < 0.001) and males (*I*^2^ = 95.09; *p* < 0.001) (see Table 2).

### Sensitivity analysis

To further investigate the potential source of heterogeneity in the analysis of the prevalence of depression among patients with TB, we conducted a sensitivity analysis by the type of depression, the assessment instrument used to measure depression, and the quality of the included studies. When limiting the analysis to studies reported depression among MDR-TB, the prevalence of depression was found to be, 52.34%, as compared studies conducted on non-MDR-TB 43.47%, even though the variation was not statistically significant (*p* = 0.320). In our sensitivity analysis based on the instrument used to measure depression among patients with TB, the highest prevalence of depression was found as it was measured by HDRS (45.45%) and the lowest prevalence was observed as measured by PHQ-9 (38.36%). The pooled estimated prevalence of depression was 55.62% as measured by BDI and 45.52% as measured by HADS. However, the difference observed in the prevalence of depression among TB patients based on the assessment instrument used was not statistically significant (*p* = 0.073) (see Table 2).

Finally, we also conducted the sensitivity analysis based on the quality of included studies, the prevalence depression was found relatively equal for high-quality (46.06%) and moderate-quality studies (47.85%) and slightly lower for poor-quality studies (40.54%, although the difference was not statistically significant (*p* = 0.657) (see Table 2).

### Publication bias

**Fig. 3:**
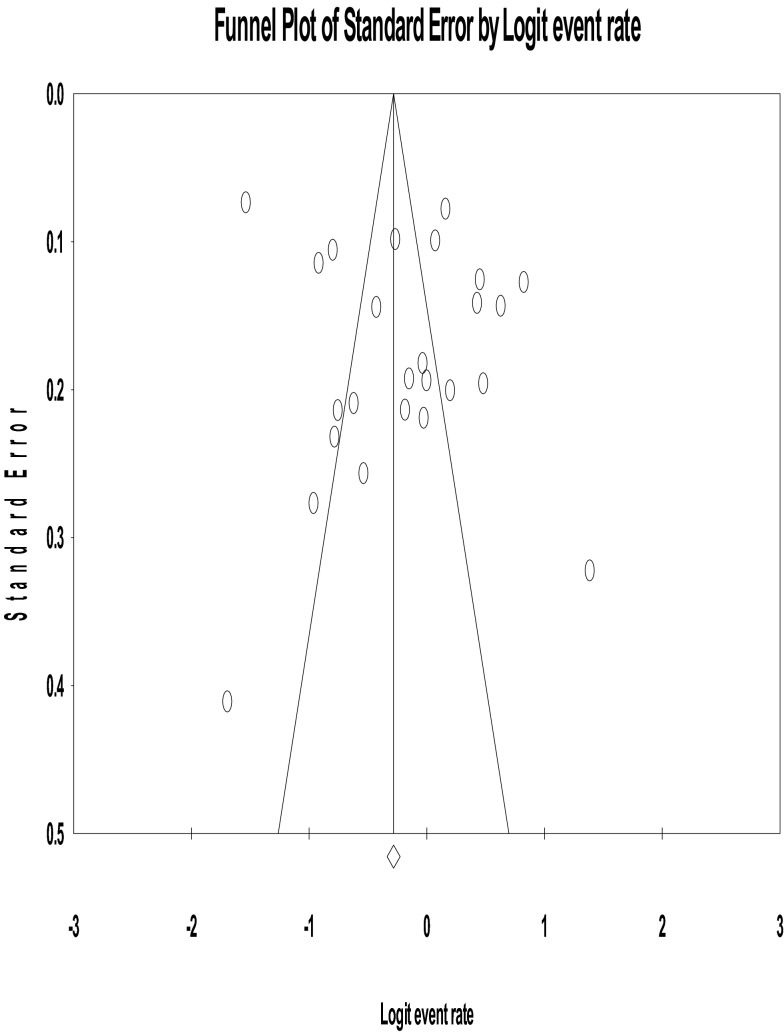
Funnel plot of standard error by logit event rate (publication bias)

The funnel plot was symmetric and Egger’s regression tests provided no evidence of substantial publication bias for the prevalence of depression among patients with tuberculosis (*B* = 2.70, SE = 2.59, *p* = 0.307) (see Fig. 3).

## Discussion

This systematic review and meta-analysis of the prevalence of depression among patients with tuberculosis showed that the pooled estimated prevalence of depression was 45.55%. This figure was remarkably higher than the estimated prevalence of depression in the general population which was 4.4% in 2017 [47]. Further, the pooled estimated prevalence of depression among patients with TB was much higher than the finding from a systematic review and meta-analysis conducted to assess the pooled estimated prevalence of depression and depressive symptoms among outpatients except TB that reported 27.0% [48]. Similarly, another systematic review and meta-analysis that assessed the prevalence of depression among patients with type 1 and 2 diabetes reported 12% and 19.1%, respectively [49]. These variations might be because chronic diseases such as tuberculosis, which are highly susceptible to perceived stigma and poor social support, may precipitate the depressive feelings and, in fact, clinical depression increases as the severity of the illness increases. This is also supported by evidence that suggested individuals with chronic medical illnesses, such as tuberculosis, may show much higher rates of prevalence of depression when compared to other non-chronic medical illnesses that range between 25 and 33% [47, 50]. Further, evidence suggests that tuberculous infection may precipitate depression or depressive symptoms in individuals due to inflammatory response and dysregulation of the hypothalamic–pituitary–adrenal axis [51]. The dysregulation of the hypothalamic–pituitary–adrenal axis has been suggested in the pathophysiology of mood disorders including depression [52].

In our subgroup analysis, the pooled prevalence of depression among MDR-TB patients was found to be 52.34% which was slightly higher than the prevalence of depression among non-MDR-TB patients (43.74), nonetheless, the variation between the observed prevalence among the two groups was not statistically significant. A recent systematic review and meta-analysis was conducted in 2018 also showed that the estimated pooled prevalence of depression among patients with MDR-TB was 50% before initiation of the medications [20]. Further, this is also complemented by a finding from the Swedish Population-Based Cohort study that revealed the severity of depression increases as the severity of the illness increases [53]. These variations might be due to the severity of MDR-TB, the effect of using multiple drugs as well as lack of hope due to stressful life which might precipitate the progression of depression among patients with MDR-TB. Further, the higher rates of depression found among patients with MDR-TB may be explained by the negative psychological impact of being diagnosed and managed for MDR-TB.

We also conducted a subgroup and sensitivity analysis on the prevalence of depression among TB patients based on the instruments used to measure depression, in this analysis the highest magnitude of depression was reported as it was measured by HDRS (61.09%) and the lowest prevalence was reported when it was measured by PHQ-9 (37.71%). The estimated prevalence of depression was 55.6% and 46.5% as it was measured by BDI and HADS, respectively. However, our test of significance revealed that the observed difference in the magnitude of depression across different rating instruments was not statistically significant (occurred by chance) (*p* = 0.07); suggesting the use of different instruments had no significant impact on the overall pooled prevalence of depression among TB patients. The highest prevalence of depression among TB patients was observed when studies using HDRS in comparison to other instruments. This might be due to most of the studies that used HDRS to assess depression were included only MDR-TB patients. The other possible reasons for the observed variation in the prevalence of depression across the various instruments include the difference in the psychometric properties across the instruments as well as the use of different cut-off points (criteria) to define depression. For example, a study conducted to assess the psychometric comparison of PHQ-9 and HADS-D for measuring depression severity in primary care showed that both instruments differed significantly in categorizing the severity of depression [54]. Further, this is also supported by another study conducted to compare psychometric properties of the PHQ-9 and BDI-II for measuring of depression which reported that the BDI-II and PHQ-9 demonstrated adequate reliability and validity in a similar way, but differed in labeling the severity of depression or depressive symptoms in patients with medical illnesses such as tuberculosis [55].

The prevalence of depression was higher in women (51.54%) when compared to men (42.25%). This is line with the result of a systematic review that assessed the gender disparity in the prevalence of depression among the patient population revealed being male sex was 63% less likely to develop depression when compared to being female sex [56]. The Global Burden of Disease (GBD) study also showed that the global annual prevalence of depression among women and men was 5.5% and 3.2%, respectively, in 2010, suggesting a 1.7-fold greater risk in women [57]. For instance, findings from epidemiological studies on adults also showed that women have higher rates of the depressive disorder compared to men, suggesting on average, the ratio is 2:1; however, this ratio is not valid for all settings and substantially varies across different settings [58, 59]. A systematic review and meta-analysis was conducted in 2017 synthesized data from 95 studies representing 1,922,064 people in over 90 different countries [60]. This review found a statistically significant association for the gender difference in diagnoses of depression, with OR = 2.37 at the age of 12 years. This suggests that women are more likely to have depression and depressive symptoms when compared to men. Further, evidence from the epidemiological studies also suggested that the gender differences in socialization may play a significant role in the higher prevalence of depression as females socialized to be more nurturing and sensitive to the opinions of others when compared to males [61, 62]. Moreover, a meta-analysis performed to evaluate gender differences in adults also reported that females tend to use a more emotion-focused and think about their problems over in their minds and this, in turn, may increase the vulnerability of females in developing depression when compared to males [63]. Selective preventive interventions may be warranted to reduce depression in women [64, 65].

## Strength and limitations

Strengths of the present study are the use of predefined search strategy, conducting data extraction and quality evaluation by two independent reviewers to minimize the possible reviewer bias. Performing sensitivity and subgroup analysis based on the severity of tuberculosis, an instrument used to assess depression, the gender of the study participants and the quality of the studies were among the strengths of the current study as well. The limitations of the study include a small number of studies that were used in our subgroup analysis which may reduce the precision of the estimate. Further, the majority of the studies were conducted in low- and middle-income countries. This may suggest tuberculosis is an underestimated problem in high-income Western countries and probably depression in TB patients is even more underestimated in Western countries.

## Conclusion

In summary, the results of the current study indicated that the prevalence of depression among patients with tuberculosis was relatively higher. We also found a higher rate of prevalence of depression among MDR-TB than non-MDR-TB patients. Further, women experience depression at higher rates when compared to men. However, the absolute reasons for the observed gender difference in the prevalence of depression need further investigations. The findings also suggest that attention needs to be given to the early screening and management of depression to alleviate this suffering. Further, given the high prevalence of depression among patients with tuberculosis, counseling and routine social support should be an integral component of tuberculosis care and management programs. Moreover, the integration of tuberculosis program with regular psychiatry services may substantially reduce the burden of depression.

## Data Availability

All data generated or analyzed during this study are included in this article.

## Abbreviations

BDI: Beck’s Depression Inventory
HADS: Hospital Anxiety and Depression Scale
HDRS: Hamilton Depression Rating Scale
MDR: Multiple drug resistance
PHQ-9: Patient Health Questionnaire-9
TB: Tuberculosis
WHO: World Health Organization
